# Report of the Medical Director of the Savannah Sanitary Association

**Published:** 1890-04

**Authors:** J. C. LeHardy

**Affiliations:** Medical Director S. S. A., Savannah, Ga.


### Southern Medical Record.

A MONTHLY JOURNAL OF MEDICINE AND SURGERY.

VOL. XX. ATLANTA, GA., APRIL, 1890. No. 4.

### Original Articles.

### REPORT OF THE MEDICAL DIRECTOR OF THE SAVANNAH SANITARY ASSOCIATION.

### UPON THE EXCESSIVE DEATH RATE AMONG THE NEGROES AND ITS INJURIOUS INFLUENCE UPON THE GROWTH OF SOUTHERN CITIES, WITH SUGGESTIONS TO CORRECT THE EVIL.

BY J. C. LE HARDY, M.D., MEDICAL DIRECTOR S. S. A., OF SAVANNAH, GA.

There is perhaps nothing which so advances the prosperity of a city as the reputation of being a healthy place, where strangers may resort without danger of being stricken down with disease, and where the chances of enjoying a long life are good. For this reason it is, that cities which are desirous of increasing their population, make strenuous efforts to prove that their own mortality is the lowest in the country. They publish monthly or weekly reports of their own deaths in parallel columns with those of other cities, and these quasi statistical tables are scattered far and wide all over the land.

In such comparisons Southern cities are necessarily at a disadvantage because the negro population is much greater at the South than at the North or West, and because negroes are so thriftless and so careless of sanitary observances that the mortality among them is more than double that among the whites.

Recognizing this fact, Savannah and other Southern cities are careful to separate the negro deaths in their periodical reports. But in the comparative statements sent out by their rivals, this separation is ignored; the deaths among the two races are added together and the general average is given as presenting the correct conclusion.

We cannot prevent this consolidation, and so long as other cities will persist in making it, we are compelled to submit to the losses which it entails upon us, and must, as remedy, devise some plan by which the mortality among the negroes can be diminished.

The peculiarities of these days' negro have led many, even at the South, to believe that it is impossible to reduce the mortality of his race to that of the white, but on inquiry into the state of affairs existing before the war, when negroes were slaves, and it was to the pecuniary interest of owners to take care of them as property, will show that this is not the fact. Slaves had healthy quarters, wholesome food was provided, the abuse of ardent spirits was prevented, personal cleanliness was required, marriage was insisted upon, regular hours for sleep and work were kept, and the settlements were carefully policed. Lying in women were tenderly looked after by the mistress or by the overseer's wife and the children were nursed under a most careful management. In case of sickness the best medical attention was procured. In fact all that could be done through sanitary measures was done to prevent sickness and death.

Those among the medical profession who had charge of negro slaves, can easily remember how healthy and sturdy they were; how prolific, when living in healthy localities, how small the mortality among them, how every plantation could boast its aged patriarchs.

In the records of mortality kept in Savannah before the war we find the ratio of death among the negro to be smaller than that among the whites. How is it then that the relative mortality of the races has been so completely reversed since the war?

The steady progress made in sanitary work within the city, and the thorough drainage of the low lands surrounding it, have had the effect of diminishing sickness and the death rate among the whites in a most remarkable manner, but if any such effect has been produced upon the negroes it certainly has been offset by other causes. The removal of the wholesome restrictions placed upon them while slaves, has left them free to go back toward the natural propensities of the race and to drift into bad habits.

At the end of the civil war, when the Federal armies overran the Southern coasts, the negroes left their comfortable homes to follow the army without provisions, without clothing or shelter, they soon gathered pell-mell in great number. Want, misery and hunger led them to steal and to perform the most abject services.

The intermingling of the sexes without restraint, led to the most revolting lasciviousness. Syphilis became dreadfully prevalent; small pox broke out among them with fearful ravages. Exposure to the weather, disease and the lack of proper food depleted their vital powers and as a sequence, the generation which followed emancipation was weakly and worthless. Lung diseases of all forms, especially consumption, which was almost unknown among them in slavery, appeared among them and to this day constitute the most prolific cause of mortality among adults.

Although the substitution of order for the chaotic condition which immediately followed the war, has brought the negroes under peaceable subjection to the laws of the State, yet they have not profited by this step toward civilization as they might have done. The observance of the laws of health cannot be enforced by existing public laws, and the negroes following their proclivities lead a life which insures the detriment of their own health and that of their white neighbors.

The greater facilities for making a living in large cities have attracted them around these centres of population. Here they live together in small houses, in crowded neighborhoods, which are necessarily filthy and unhealthy, and here they lead a life well calculated to produce the large percentage of mortality which we find to occur among children and young adults, and the surprising number of still and premature births.

While the conditions under which white and negro laborers live, in this city, are very similar, and their wages are the same, it is unquestionable that the negro mortality is much the greater, and this excess can only be attributed to their mode of life.

The negro is not provident, he lives from hand to mouth, spending the best part of his wages before they are earned. Irregular in his eating, he usually gorges himself at night, goes to his work on an empty stomach and bolts a snack at noon.

Not being aware of the danger of eating unsound food, he buys his meat and vegetables where he can get them cheapest, whether they are good or spoiled, and generally they are not fit for food.

Sociable in his nature he spends the best part of the night in drinking and carousing, or if religiously inclined, in shouting and praying, thus depriving himself of the necessary amount of sleep and acquiring habits of licentiousness.

The necessity for making a living forces the father and mother of the family to work out, and the children are left in the care of other children, or of old women; they are stuffed with coarse and unwholesome food,badly clothed and kept filthy in their persons and surroundings. This treatment causes diarrhœal diseases and marasmus to which most of the great excess of mortality during the first year of life is due.

Sleeping in crowded and ill ventillated rooms, exposure to the weather, loss of rest, improper food, inherited syphilis, etc., account for the great excess in mortality from consumption and pneumonia.

Bad management and neglect of mother and child by ignorant midwives, together with foul and badly ventilated rooms for the excess in trismus. Neglect to call a medical man and bad nursing for the excess in deaths, without physician in attendance, 7 whites, 182 blacks. Bad ventilation, over crowding, foul air, improper food, lazy habits cause excess from dropsy.

Comparing the present condition and mortality of the negroes, with their condition and mortality under slavery, it seems to me that much could be done to diminish sickness and death among them, by proper sanitary regulations, which municipal authorities have power to make and inforce. Ordinances should be passed regulating the building of small houses, prescribing the area to be left unbuilt upon each lot, as air space for each house, requiring the ventilation of every sleeping room, interdicting the building of close fence around yards, and preventing the accumulation of foul matter by abolishing privy vaults and subitituting the pail or other system. Yards and houses should be carefully and regularly policed to prevent the accumulation of animal and vegetable matter. The eating of unwholesome food can certainly be prevented by officers whose duty should be to inspect all meat, fish, vegetables, groceries and provisions of all kind offered for sale, and having power to destroy every unsound article, and to bring the venders before the authorities for punishment.

All alcoholic beverages offered for sale should be inspected and destroyed if found adulterated. Some officer should be appointed to investigate each case of death when no physician was in attendance in order to bring the responsible party to justice when ever willful neglect can be discovered.

All medicines should be required to pass an examination before a board appointed for the purpose by the authorities; any one practicing without a license from this board should be severely punished.

Houses of ill fame should be placed under strict regulations ; the women should be registered, licensed and regularly examined. Any one plying the trade without license should be made to work for the county.

Through the moral influence of our sanitary association and with the help of the Doctors and of the Clergy, men and women could be instructed in the knowledge of the sanitary needs of the family, how to take care of themselves, to feed and clothe children and to nurse the sick. They should be made to appreciate the value of wholesome food, of regular habits and of cleanliness.

Were such measures as these adopted by our City Council and by this association their effect would prove salutary to the whole population, but more particularly to the colored portion of it, for it is that portion which especially requires them.

There is one thing more relating to this subject to which I wish to call attention. It is a common thing for cities in order to prove a low death rate, to overshoot the mark, and reduce this rate below possibilities. Some have even brought the ratio below 10 in 1,000 inhabitants, which would give an average life of over 100 years. Savannah in the Mayor's report for 1888 gives 11:45 in 1,000, of the mortality rate among the whites—or an average life of 87 1-3 years, that is to say, that the chances are that every inhabitant of this city will live to that age—an absurdity.

